# Continuous patient state attention model for addressing irregularity in electronic health records

**DOI:** 10.1186/s12911-024-02514-2

**Published:** 2024-05-03

**Authors:** Vinod Kumar Chauhan, Anshul Thakur, Odhran O’Donoghue, Omid Rohanian, Soheila Molaei, David A. Clifton

**Affiliations:** 1 Institute of Biomedical Engineering, University of Oxford, Old Road Campus Research Building, OX3 7DQ Oxford, UK; 2 Oxford-Suzhou Centre for Advanced Research, Suzhou, China

**Keywords:** Deep learning, Neural ordinary differential equations, Irregular time series, Electronic health records, Perceiver, In-hospital-mortality, MIMIC-III

## Abstract

**Background:**

Irregular time series (ITS) are common in healthcare as patient data is recorded in an electronic health record (EHR) system as per clinical guidelines/requirements but not for research and depends on a patient’s health status. Due to irregularity, it is challenging to develop machine learning techniques to uncover vast intelligence hidden in EHR big data, without losing performance on downstream patient outcome prediction tasks.

**Methods:**

In this paper, we propose Perceiver, a cross-attention-based transformer variant that is computationally efficient and can handle long sequences of time series in healthcare. We further develop continuous patient state attention models, using Perceiver and transformer to deal with ITS in EHR. The continuous patient state models utilise neural ordinary differential equations to learn patient health dynamics, i.e., patient health trajectory from observed irregular time steps, which enables them to sample patient state at any time.

**Results:**

The proposed models’ performance on in-hospital mortality prediction task on PhysioNet-2012 challenge and MIMIC-III datasets is examined. Perceiver model either outperforms or performs at par with baselines, and reduces computations by about nine times when compared to the transformer model, with no significant loss of performance. Experiments to examine irregularity in healthcare reveal that continuous patient state models outperform baselines. Moreover, the predictive uncertainty of the model is used to refer extremely uncertain cases to clinicians, which enhances the model’s performance. Code is publicly available and verified at https://codeocean.com/capsule/4587224.

**Conclusions:**

Perceiver presents a computationally efficient potential alternative for processing long sequences of time series in healthcare, and the continuous patient state attention models outperform the traditional and advanced techniques to handle irregularity in the time series. Moreover, the predictive uncertainty of the model helps in the development of transparent and trustworthy systems, which can be utilised as per the availability of clinicians.

## Background

Electronic Health Records (EHR) often contain irregular time series (ITS) data due to uneven time intervals between measurements of patient attributes [1]. In EHR, ITS can occur due to several reasons. For example, data in an EHR system are not recorded for research purposes but are recorded as per guidelines, medical requirements and for supporting medical claims etc., and all measurements and treatments depend on a patient’s health status [2]. Since machine learning algorithms mostly work with fixed-size feature vectors so this irregular data is converted to regularly spaced data, which leads to the generation of missing values for intervals when no measurements were taken [1, 3]. ITS are widely prevalent in primary and secondary care, including critical care, e.g., the MIMIC-III dataset has a missing rate of over 90% for hourly sampled ITS [1, 4, 5].

The adoption of EHR in healthcare has resulted in big data that has presented great opportunities for the development of machine learning algorithms and artificial intelligence technologies to reduce the burden on the healthcare system, support clinical decision making and efficient management of healthcare resources [6–16]. Machine learning algorithms, however, are primarily predicated on an assumption of coherent fixed dimensional feature vectors, and presence of ITS in EHR invalidates that assumption. The irregularity presents challenges in utilising the vast intelligence hidden in EHR big data and developing machine learning algorithms without losing performance on downstream tasks [17]. As a result, it is important to develop techniques for appropriately handling irregularity in EHR for numerous reasons, such as resource management, triaging, diagnosis, treatment, and prognosis. Due to the significance and widespread presence of irregularity in healthcare, ITS and the resulting missing values have received great attention from the research community, and several approaches have been proposed to address the irregularity [2, 3, 18–23]. For example, from the traditional statistical techniques for replacing missing values (such as using zero and mean values), imputation, interpolation, and matrix completion-based techniques [24] to advanced techniques, including neural processes [2], adaptation of recurrent neural networks (RNN) [25], neural ordinary differential equations (NODE) based RNN [18], and attention-based techniques [19] (please refer to [26] for a recent review).

Traditional basic statistical techniques for replacing missing values, such as using zero, mean, median, and carry-forward, are biased and rely on underlying assumptions about how data are generated. This is reported to result in a loss of performance in patient outcome predictions [27]. Many recent techniques for addressing irregularity fall short of capturing feature correlations in data [25], consider missingness and patient outcome predictions separately [28], and fail to learn the pattern of missingness, or are ineffective in handling long sequences and noise [24] etc. While some techniques, like [18], can handle completely missing time-step, most techniques, like [3], can only handle partially missing values.

In this paper, our contributions are two-fold: first, we propose a computationally efficient variant of transformer [29] based on the idea of cross-attention [30–32], called Perceiver, to process long sequences of time series in EHR, and second, we propose a continuous variant of these attention-based models, i.e., Perceiver and transformer to address the above limitations of ITS techniques. The proposed continuous patient state attention models learn patient health trajectory for end-to-end learning from observed time steps, which can consider long sequences, noise, and completely missing time steps, including sparse time series.

The transformer-based models are one of the most successful deep learning techniques, which demonstrated impressive results across different domains [3, 33, 34]. However, the quadratic dependence of the transformer-based models on input limits their application to long sequences. To address this issue, recently, cross-attention-based models [30–32] are proposed to squeeze large inputs to tighter learnable latents, which are then followed by self-attention operations (transformer) on squeezed inputs. Inspired by the idea of cross-attention, we develop a Perceiver model for EHR to handle long sequences of time series, as discussed in Methods section. Perceiver could be useful in healthcare since EHR data represent a lot of information about patients, and working with a complete and long trajectory of a patient’s health status would yield better results.

To address irregularity in EHR data, we propose continuous variants of Perceiver and transformer for patient’s health status, called as COntinuous Patient state PERceiver (COPER) and Continuous Transformer (CTransformer), respectively. These continuous state attention models learn patient health dynamics, i.e., patient health state trajectory from the observed irregular time steps from which patient health state can be sampled at any time-step and used to generate a regular time series to be processed with Perceiver/transformer model. COPER/CTransformer can handle completely missing time steps, i.e., time steps where no data is recorded, as well as small noise in the observations because it can generate the complete time series after learning from the observed irregular time steps. COPER/CTransformer achieves continuity in patient’s health status using embedding and NODE. The modelling of a patient’s health status could be helpful in several tasks, including treatment, prognosis, diagnosis and disease progression modelling.

The proposed work has some similarities to [18, 35] and [30–32]. The work in [18, 35], specifically [18] proposed latent ODE (LODE) based on recurrent neural networks and developed an encoder and decoder-based architecture employing NODE in both to address irregularity. LODE learns the dynamics of the hidden state of the model. Thus, our work differs from LODE, in terms of using non-recurrent neural networks, using one NODE, and using NODE for continuity of patient state rather than the hidden state of the neural network. Moreover, the works in [30–32] are based on the idea of cross-attention of inputs with learnable latents for reducing the complexity of the transformer-based architectures. Our work is also inspired by the idea of cross-attention but is architecturally different (refer to Methods section for details) from the existing work and presents an application to solving a different problem, i.e., irregularity in EHR.

To evaluate the empirical performance of the proposed techniques, we have used in-hospital-mortality (IHM) prediction task using MIMIC-III and PhysioNet-2012 datasets, which contain time series data from the intensive care unit (ICU). Area under the receiver operating curve (AUROC) and area under the precision recall curve (AUPRC) are used as performance metrics. Perceiver is compared with long short-term memory (LSTM) [36], temporal convolutional network (TCN) [37] and DLinear [38] as baselines. For evaluating the performance of the continuous patient state models, we have designed experiments to study irregularity at 25%, 50% and 75% missing time steps by randomly removing the time steps, and compared them with simple baselines, like LSTM and Perceiver with carry forward, as well as advanced state-of-the-art techniques, like LODE [18], Multi-Time Attention Network (mTAND) [19] and DLinear [38].

The contributions of the paper are summarised below.

- A computationally efficient cross-attention based variant of transformer, called Perceiver, is proposed for time series in EHR data. The cross-attention operation helps to reduce the computations by squeezing long sequences to smaller latents.
- To address irregularity in EHR data, we propose continuous variants of Perceiver and transformer, called COPER and CTransformer, respectively, which learn the dynamics of a patient’s health from irregularly observed time steps using neural ordinary differential equations.
- Empirical evaluation of the proposed techniques is performed on in-hospital-mortality prediction task using MIMIC-III and PhysioNet-2012 datasets. The experiments show that Perceiver can be used as a potential alternative for processing time series in EHR, and reduces the computations by around nine times as compared to the transformer without significant loss of performance. The carefully designed experiments for continuous patient state models also show their efficacy in dealing with irregular time series in EHR. Moreover, the proposed techniques also employ predictive uncertainty to improve performance, transparency and trustworthiness.

The preliminary idea of this work was published in [39]. This paper revises the idea and the empirical evaluation of [39] in several ways: (i) the architecture/idea is revised to have continuity only in patient health state as this suffices to address irregularity in EHR, which also improves results than having continuity in input as well as output spaces. Moreover, similar to continuous Perceiver, a continuous transformer is also proposed, (ii) methodology is discussed in detail and algorithmic details are also provided, (iii) new experiments are added to show the utility of Perceiver over transformer architecture, (iv) additional dataset and metric are considered for evaluating the proposed techniques, and (v) experiments are added to utilise the uncertainty of the proposed techniques to improve transparency and hence trustworthiness.

## Methods

In this section, we discuss the architecture and algorithmic details of the proposed Perceiver and the continuous patient state models.

### Perceiver

Transformer [29] based models have been successful across different domains with different modalities, including time series in healthcare [8, 40]. However, the main limitation of these models is their quadratic dependence on the input size, which results in large computational complexity when dealing with long context inputs – limiting their applicability to such problems that are quite common in healthcare time series data [41].

The cross-attention-based models [30–32] are recent advancements to transformer [29] based models and they address issue of a quadratic dependence of transformers on input by introducing a cross-attention operation of learnable smaller latents with inputs. The cross-attention distils a long sequence input to smaller latents which is followed by self-attentions (transformer) on the squeezed latents, as given below. In our time series setting, a long sequence of time steps can be squeezed into a customised number of latents for processing with transformer based models, which otherwise could be computationally very expensive or even infeasible in some cases to use transformers directly on input data.

**Fig. 1:**
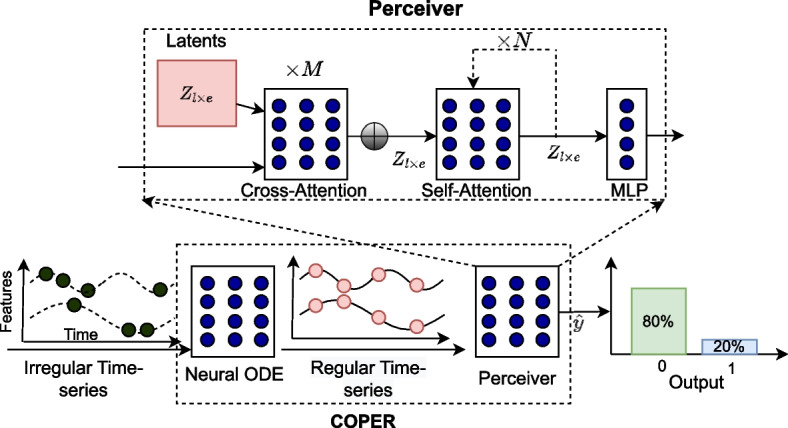
Architecture of COPER: An embedding of irregular time series is passed through NODE, which captures patient health dynamics from observed time steps, and is used to generate a regular time series. The generated regular time series is then fed to Perceiver model which first squeezes the long sequence of *T* time steps to a $l<T$ latents using cross-attention and then followed by self-attention operations

The architecture of COPER model, and Perceiver as a component of COPER, are presented in Fig. 1. The proposed Perceiver model borrows the idea of cross-attention from [30–32] but has different architecture as shown in Fig. 1. Perceiver uses a cross-attention operation to squeeze the input sequence length from *T* time steps into $l<T$ number of latents of the same feature dimension as the original sequence. The cross-attention is applied *M*-times on the input and the outputs are averaged, which are then processed using transformer (self-attention) layers, leading to lower computations as compared with processing the original input directly with transformers.

Suppose $\lbrace X_i, y_i\rbrace _{i=1}^n$ be a training dataset with *n* patients where $X_i \in \mathbb {R}^{ t_d \times D}$ represents ITS of a patient *i* having *D* features for uneven $t_d$ time steps recorded for feature *d*, where each time-step represents health status of the patient. $y_i \in \lbrace 0, 1 \rbrace$ represents patient outcomes (say in-hospital-mortality, where 0 refers to a patient, who lives to be discharged, else dies in hospital). First, let us define attention operation [29], which is a scaled dot product attention between a set of queries (*Q*), keys (*K*) and values (*V*), as given below.

1 $$\begin{aligned} \alpha (Q, K, V) = softmax \left( \frac{QK^T}{\sqrt{d_k}}\right) V, \end{aligned}$$

where $\alpha$ denotes attention function and $d_k$ is dimension of key vector. The self-attention operation has $Q=K=V=X_i$ while cross-attention operation has $Q=Z \text { and } K=V=X_i$, where $X_i \in \mathbb {R}^{ T \times e}$ represents a data point (a patient in our case) with *e* features having *T* time steps, and $Z \in \mathbb {R}^{ l \times e}$ for $1 \le l \le T$ number of latents. Algorithm 1 provides details about flow of information through COPER and Perceiver, which is explained in the next subsection.

### Continuous patient state attention

Continuous patient state attention models are advanced deep learning models to handle irregular time series data in EHR. They learn a patient’s health trajectory from observed time steps, i.e., observations of the patient’s health status at uneven time steps. By learning patient health dynamics, they can handle irregularity as well as noise in the patient’s health status for successfully predicting patient health outcomes.

COPER is based on the recent advancements of neural ordinary differential equations (NODE) and cross-attention-based models to handle ITS in EHR and can be applied to different tasks. The overall architecture of COPER model is represented in Fig. 1 and the pseudocode for representing the flow of information is given in the Algorithm 1.

**Figure:**
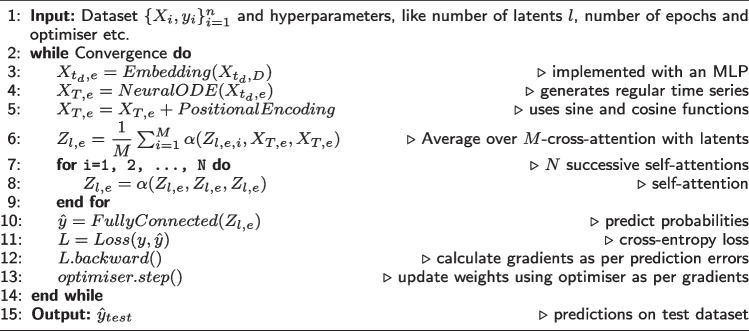
**Algorithm 1** COntinuous Patient state PERceiver

As described in the algorithm, COPER processes input ITS $X_{t_d,D}$, having *D* features with $t_d$ time steps for feature *d*, first by learning an optional embedding $X_{t_d,e}$ of size *e* for each time-step, using a single layer multi-layer perceptron (MLP). These embeddings are then processed with NODE, which is another recently proposed category of neural networks. NODE learns the dynamics of a patient’s health status from which a patient’s health status can be inferred at any time and a regular time series can be generated. NODE consists of a neural network and a black-box ordinary differential equation (ODE) solver. The neural network outputs a derivative of the patient’s health status, which is fed to an ODE solver. The ODE solver enables the model to calculate the patient’s health status at any time step and hence enabling it to address ITS, as described below.

2 $$\begin{aligned} \begin{array}{l} \frac{dZ}{dt} = f_\theta \left( Z(t), t \right) ,\\ Z_0, ..., Z_N = \textsc {ODESolver}\left( f_{\theta }, Z_0, \left( t_0, ..., t_N \right) \right) , \end{array} \end{aligned}$$

where *Z* is a patient state, $f_\theta$ is a neural network which parameterises the derivative of the patient state. The ODESolver takes the derivative from $f_\theta$ and initial patient state $Z_0$ and calculates the patient’s health status at desired time steps $\left( t_0, ..., t_N \right)$.

Steps 5-10 are part of Perceiver model, which first adds positional encoding to the input for maintaining the order information of time steps using sine and cosine functions, as given below [29]:

3 $$\begin{aligned} PositionalEncoding_{(pos, 2i)}{} & {} = sin(pos/ 10000^{2i/d_{model}}),\nonumber \\ PositionalEncoding_{(pos, 2i+1)}{} & {} = cos(pos/ 10000^{2i/d_{model}}). \end{aligned}$$

As shown in Fig. 1, Perceiver applies *M* cross-attentions on encoded input with latents *Z*, followed by *N* successive self-attention operations on average of cross-attention operations ($M=1, N=1$ in our experiments). The resulting output is then passed through a fully connected layer to predict output probabilities and followed by a standard machine learning process to update parameters in an end-to-end differentiable manner.

An architecture and algorithm for CTransformer can be obtained by replacing cross-attention operation with self-attention in the architecture and algorithm of COPER.

## Results

This section presents details about a prediction task, datasets, performance metrics, baselines, and experiments.

### Datasets and baselines

The proposed models are evaluated for in-hospital mortality (IHM) prediction task using two publicly available datasets, i.e., PhysioNet Challenge 2012 dataset (hereon referred to as PhysioNet) [42, 43] and Medical Information Mart for Intensive Care (MIMIC-III) dataset (hereon referred to as MIMIC) [44]. These are time series datasets based in the intensive care unit (ICU) setting. IHM is a binary classification problem that determines whether a patient will survive their hospital stay or pass away within the first 48 hours of ICU admission using hourly data. IHM prediction is particularly crucial for resource management, triage, initial risk assessment, and creating successful treatment programmes [25]. For preprocessing of MIMIC dataset, we have followed [45] to get a dataset with 76 features and 14,681, 3,236 and 3,222 samples in train, validation, and test datasets^1^, respectively. For PhysioNet dataset, we follow preprocessing as used in [18]. The dataset has 47 features and a total of 8,000 samples. Due to the smaller size, we have used 5-fold cross-validation in our experiments. Furthermore, validation data is taken as 20% of the training data.

Two sets of experiments are designed as follows: the first set presents Perceiver model – a computationally efficient variant of transformer – as a potential alternative for learning from time series data. The experiments compared the model with Long Short-Term Memory (LSTM) [36] and Temporal Convolutional Network (TCN) [37] – the widely used techniques for handling time series data, and DLinear [38]. Experiments are also designed to show how the latents in Perceiver can be used to squeeze long sequences into a tight smaller number of latents to reduce computational cost. The second set of experiments presents continuous patient state attention models for handling irregularity in EHR. Since our proposed work is based on attention and NODE so for comparative study, we have chosen simple baselines as well as baselines based on state-of-the-art attention and NODE-based techniques. The selected baselines are LSTM and Perceiver with carry forward to deal with missing steps, and Multi-Time Attention Network (mTAND) [19], and latent ODE (LODE) [18] which are advanced state-of-the-art techniques for handling irregularity and are based on attention and NODE, respectively. We also consider DLinear [38] which is inspired by Autoformer [46] and FEDformer [47] and decomposes input data into trend and seasonal components for carrying out different downstream tasks, like classification, forecasting and imputation, as available from TSlib^2^ [48]. To study irregularity, we have designed experiments at 0%, 25%, 50% and 75% missingness by randomly removing time steps. The area under the receiver operating curve (AUROC) and the area under the precision-recall curve (AUPRC) are used as a performance metric for the comparative study.

### Experimental settings

Hyperparameters of COPER are selected using a random search, and trial and error over a range of values: embedding layer is implemented using a multi-layer perceptron (MLP) with a single hidden layer of 32 (16, 32, 64, 128) neurons (where values inside parenthesis represent the set of values tried), NODE is implemented using an MLP with three hidden layers of 128 (50, 100, 128) neurons for each NODE, cross- and self-attention heads have 128 (32, 64, 128, 256) dimensions, latents have 64 (32, 64, 128, 256) dimensions, dropout for attentions and NODE networks are set to 0.5 (0.2, 0.3, 0.4, 0.5, 0.6). The number of latents, unless specified, are set equal to the number of time steps. The number of cross-attention operations is set to one, i.e., $M=1$ and the self-attention operations are $N=1 (1, 2, 3, 4, 5)$. For LSTM, the number of layers is set to two (one, two) each with a hidden state of size 50 (16, 32, 50, 64, 128), the dropout rate is set to 0.5 (0.2, 0.3, 0.4, 0.5, 0.6) and single-directional (single, bi). The TCN implementation and hyperparameter setting is followed from [37] with a dropout rate of 0.70%^3^. For mTAND^4^, we follow the source paper and have set the hyperparameters as (PhysioNet, MIMIC): alpha (100, 5), learning rate (0.0001, 0.0001), rec-hidden dimension (256, 256), gen-hidden dimension (50, 50), latent-dimension (20, 128), norm (true, true), kl (true, true), learn-emb (true, true), k-iwae (1, 1), and number of epochs are set to 300. For the LODE^5^, we follow the source paper and have set hyperparameters as (PhysioNet, MIMIC): latent-dimension (20, 40), rec-dimension (40, 80), Poisson (true, true), and number of epochs are set to 300. For DLinear [38], we follow implementation and hyperparameters from TSlib^6^ [48], and use 3 e-layers, 128 dimensions for d-model, 256 dimensions for d-ff 256 and a learning rate of 0.001, while loss is sum of imputation loss as well as classification loss.

For all the models, we have set Adam [49] as an optimiser with a constant learning rate of 0.0001. To avoid overfitting, in addition to dropout, we have used early stopping with the patience of 10 epochs. Total number of epochs is set to 100 unless provided by the baseline paper. The batch size is set to 64 for all the models except LODE, where it is set to 32 with the MIMIC dataset because LODE is memory intensive and the machine crashes with 64 data points. Each experiment is executed with five seed values. All the experiments are implemented in PyTorch [50] and executed on an Ubuntu machine (64GB RAM, 1 NVIDIA GeForce GPU 12 GB). The code is publicly released and verified at https://codeocean.com/capsule/4587224.

### Perceiver

**Fig. 2:**
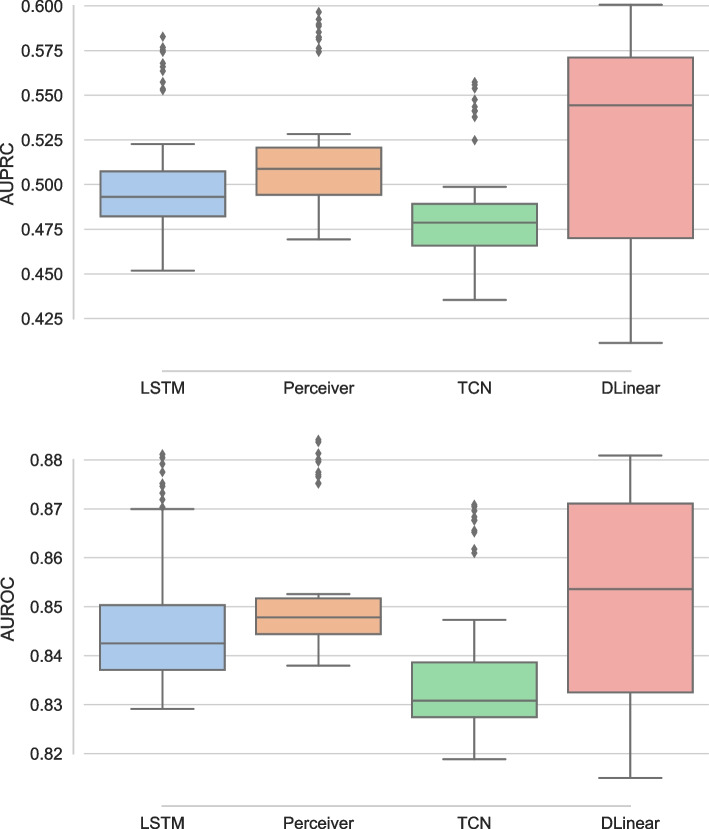
Comparative study of Perceiver against the baselines on PhysioNet dataset using AUPRC (left) and AUROC (right)

We compared the proposed Perceiver model against LSTM, TCN and DLinear. Figure 2 presents the comparative study on PhysioNet dataset using AUPRC and AUROC as the performance metrics. The left panel presents results using AUPRC, and we observe that Perceiver significantly outperforms LSTM ($p<.001$). DLinear outperforms all the baselines, however, it has larger variability in performance and it does not show significant improvement over Perceiver ($p>.05$). TCN performs the worst in terms of AUPRC on PhysioNet. The outlier performance in all the models is present due to cross-validation, as the performance of the models is better on one of the folds compared to the rest. The right panel of the figure presents results for the AUROC metric, and we observe results similar to AUPRC. We find that Perceiver significantly outperforms LSTM ($p<.001$), and TCN, once again, performs the worst. The performance difference between DLinear and Perceiver is not significant ($p>.05$). The variance in the values of Perceiver model is the least among all the models. Thus, Perceiver model exhibits either better or at par performance against the baselines on PhysioNet dataset for the in-hospital mortality prediction task.

**Fig. 3:**
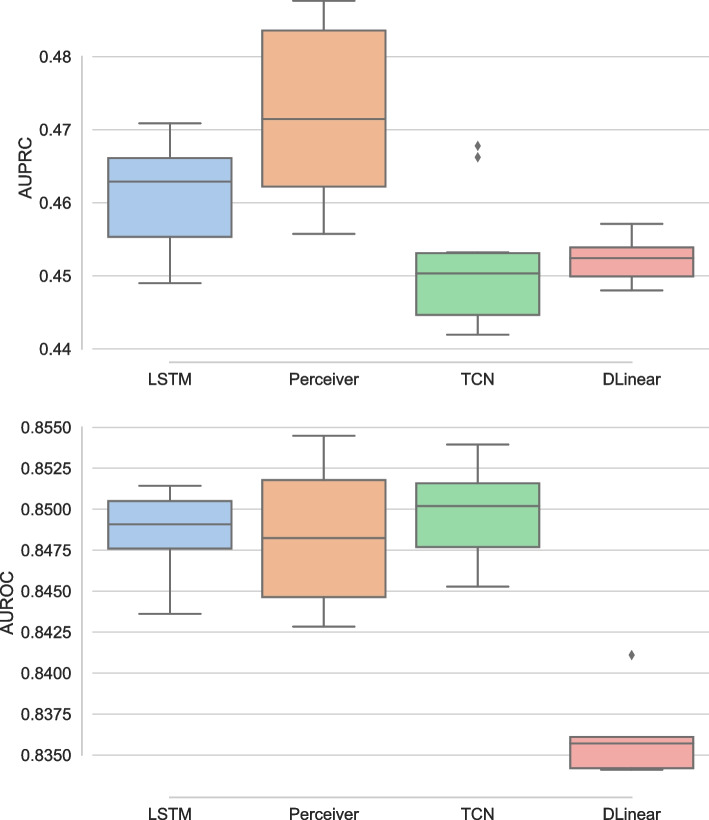
Comparative study of Perceiver against the baselines on MIMIC dataset using AUPRC (left) and AUROC (right)

The comparative study of Perceiver with the baselines on MIMIC dataset is presented in Fig. 3. The left panel compares AUPRC and as it is clear from the figure, Perceiver slightly outperforms the baselines. Moreover, as observed earlier for PhysioNet, LSTM surpasses TCN. The right panel compares the performance using AUROC and has results different from those observed with the PhysioNet as well as AUPRC on MIMIC. All the models perform very close to each other, except DLinear, as the maximum variation in the performance was around 0.01, and TCN performs the best on average. Perceiver significantly ($p<.05$) outperforms DLinear for both the cases. AUROC is similar in MIMIC and PhysioNet datasets, although AUPRC is slightly better for PhysioNet than MIMIC. The performance differences between the two datasets could be attributed to differences in the number of features, data points, and missingness.

Next, we present experiments to show the utility of Perceiver over transformer. The key idea of Perceiver is the use of cross-attention operation (Step 6 of Algorithm 1) to squeeze a long sequence to a customised smaller learnable latents. This helps Perceiver to manage computational complexity as compared with the self-attention operation of transformers, which has a quadratic dependence on input sequence and may not be suitable for large inputs.

**Fig. 4:**
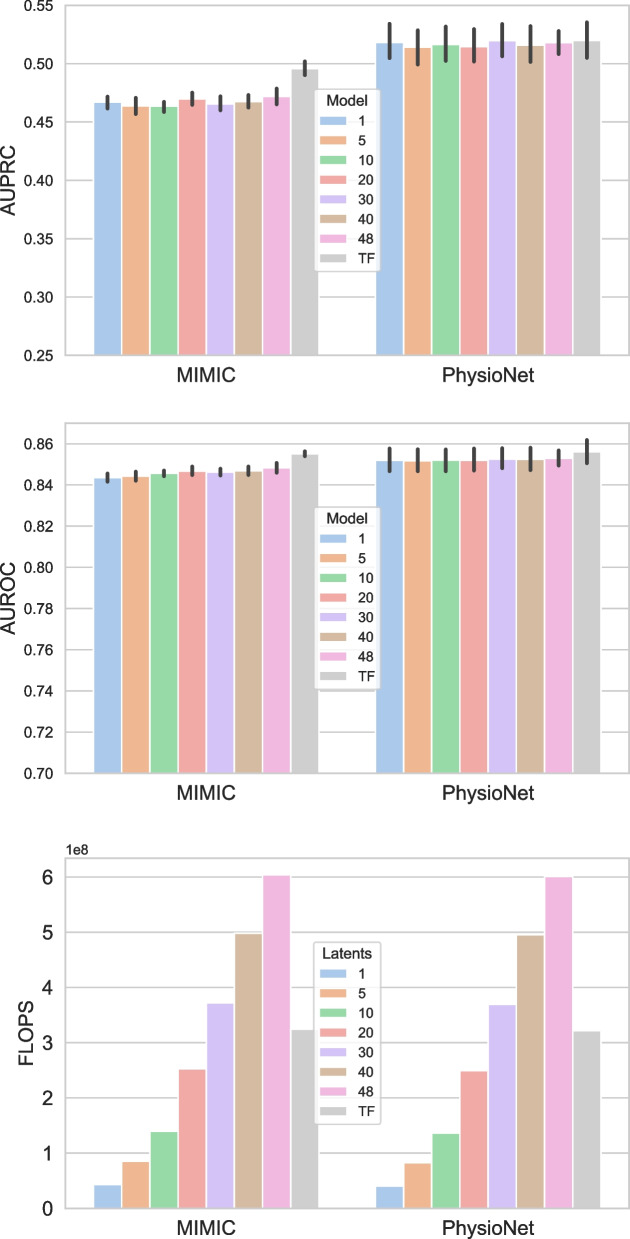
Comparative study of Perceiver with varying number of latents and transformer (TF) models, in terms of AUPRC (left), AUROC (right) and FLOPS (bottom)

Figure 4 compares computational requirements and performance of Perceiver and transformer on PhysioNet and MIMIC datasets. The bottom panel presents floating-point operations per second (FLOPS), top left and right panels compare AUPRC and AUROC, for the transformer and Perceiver with varying numbers of latents from one to length of the input, i.e., the number of time steps in the input (48 in our case). From the figure, we observe that by controlling the number of latents in Perceiver, we can reduce computations as compared with the transformer. We can reduce computations by around nine times on MIMIC and PhysioNet datasets, without any significant drop in performance except AUPRC on MIMIC dataset. Transformer and Perceiver have the same architecture except for the latents introduced by the Perceiver, and because of those latents, the Perceiver takes more FLOPS for 30 to 48 latents. The computational time complexity of a self-attention layer is denoted by $O(D.T^2)$, where *D* represents the dimensionality of the input sequence and *T* denotes its length. Similarly, for the cross-attention layer, the time complexity is *O*(*D*.*T*.*l*), with $l\; (l<<T)$ being the length of the latent vectors. In the case of Perceiver, the dimensions of the query are dependent upon the latents. Consequently, after the cross-attention layer, subsequent self-attention layers in Perceiver exhibit a complexity of $O(D.l^2)$. This characteristic enables Perceiver to effectively manage complexity through its latent vectors, facilitating the processing of inputs or sequences of considerable length. Regarding space complexity, we need to store query, key, value, and attention score matrices. So, the space complexity of the self-attention layer is represented as $O(3D.T + T^2)$. Conversely, for cross-attention layers, the complexity is $O(2D.T + D.l + T.l)$, and for subsequent self-attention layers in Perceiver, it becomes $O(3D.l + l^2)$. These complexities underscore Perceiver’s efficiency in handling both time and space requirements, contributing to its ability to mitigate computational overhead. Additionally, Perceiver improves the inference time over the transformer by up to 8% by controlling the latent dimension.

### Continuous patient state attention

Here, we present results for the proposed continuous attention models, i.e., COPER and CTransformer, to deal with irregularity in EHR data. Continuous attention models learn patient health dynamics from observed irregular observations, each of which represents a patient’s health state at a given time. Once patient health dynamics are learned, missing time steps can be sampled. To study the continuous models, we specifically design experiments at missingness of 0%, 25%, 50% and 75%, and study the performance of the proposed models against the baselines, such as mTAND, DLinear and LODE, as well as LSTM and Perceiver using carry forward techniques. In the carry forward technique for addressing irregularity, we simply replace missing steps with previously available observation.

**Fig. 5:**
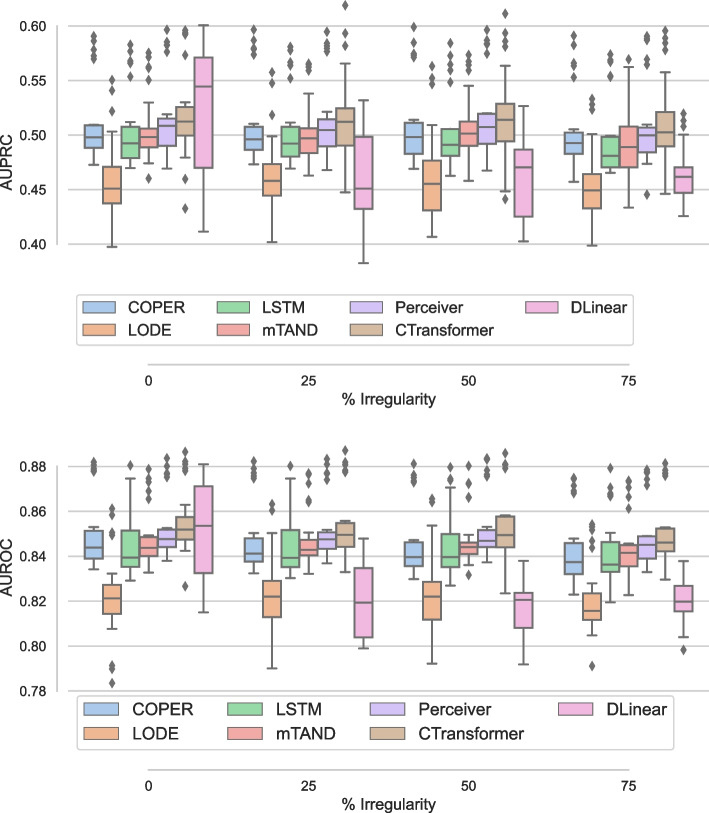
Comparative study using varying irregularity on PhysioNet dataset using AUPRC (left) and AUROC (right)

The comparative study of the proposed continuous patient state models against the baselines on the PhysioNet dataset is presented in Fig. 5 using the AUPRC (left) and AUROC (right). From the left panel, we find that CTransformer is the best model, except at no missingness where DLinear performs the best, and performs slightly better than Perceiver model. LODE performs the worst. Except for DLinear, with increasing irregularity in EHR, mostly the performance remains the same except for a slight drop at 75%. DLinear exhibits the most variation in performance and experiences the largest performance decline with 25% missing data. However, its performance stabilises after this point. This is likely due to its inability to identify seasonal and trend patterns, which are not particularly prominent in EHR data, especially with missing time steps. The right panel of the figure compares AUROC and has a performance similar to AUPRC. However, DLinear is the worst performer and again shows more variability than the rest of the models. Simple baselines with carry forward, i.e., LSTM and Perceiver, also handle irregularity quite well. However, Perceiver performs better than LSTM. This agrees with some of the literature [51] which shows that carry forward works well for EHR in some settings.

**Fig. 6:**
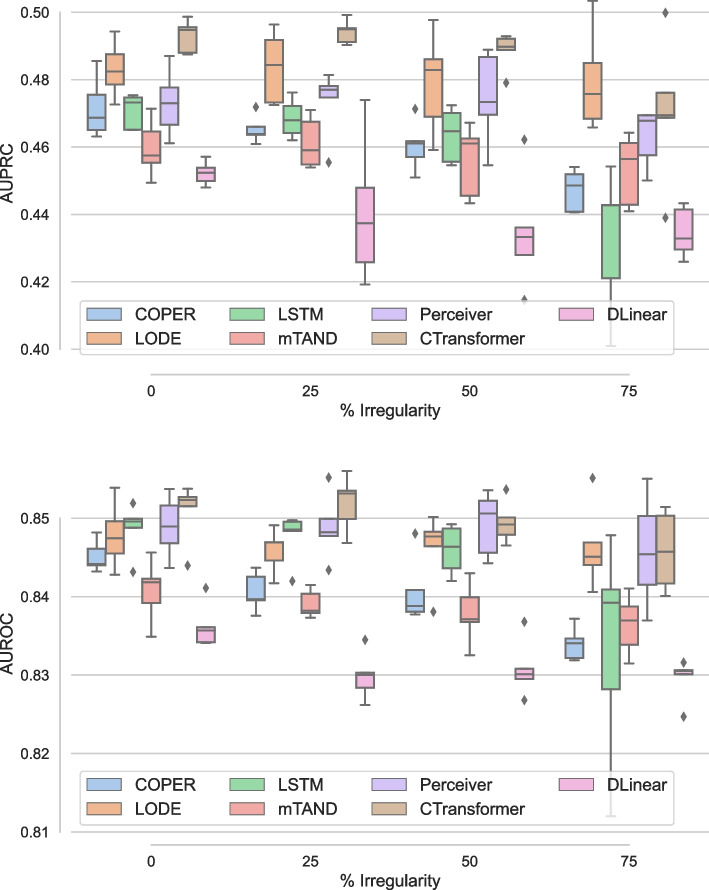
Comparative study using varying irregularity on MIMIC dataset using AUPRC (left) and AUROC (right)

Figure 6 presents the performance of different models with varying degrees of irregularity on MIMIC dataset. The performance trends on MIMIC differ slightly from PhysioNet. Overall, there is less variability in the results for all the models and the variability in performance increases on MIMIC with increasing irregularity. The proposed continuous variant of the transformer, i.e., CTransformer significantly ($p<.01$) outperforms all the baselines and shows small variability in performance. DLinear performs the worst as it has more decrease in performance with increasing irregularity. LSTM shows more variability in the performance with increasing irregularity. However, Perceiver, which also uses a simple carry forward mechanism like LSTM, performs with almost no drop in performance until 50% irregularity and a slight drop at 75% irregularity. Perceiver also performs better than its continuous version COPER. One potential reason for this could be over 90% missingness in MIMIC dataset [1]. LODE performs better on MIMIC dataset than PhysioNet dataset, as it observes a small drop in performance with increasing irregularity.

The techniques which utilise NODE for handling irregularity in EHR, i.e., COPER, CTransformer and LODE, are computationally expensive due to the use of an MLP in NODE. Among these, LODE uses two NODEs, one each in encoder and decoder, and is the most computationally expensive. For MIMIC dataset, one run of LODE can take up to two days, so it is not a good option for long sequences.

During the evaluation of continuous attention models, we have a choice of either generating the entire time series after learning the dynamics from the ITS or keeping the observed time steps and generating only the missing time steps. For lower irregularity and noisy data, generating the entire time series is helpful to reduce the effect of the noisy data, however, for higher irregularity generating only missing time steps is better.

### Ablation study

In this subsection, we present an ablation study of the proposed continuous patient state attention models. We compare the proposed model, which uses one NODE, to using two NODEs, called COPER-2ODE, as was done in the preliminary idea presented in [39]. Additionally, we replace the attention models in the proposed model with LSTM, i.e, we develop a continuous patient state based LSTM, called LSTM-ODE where NODE learns patient health dynamics and addresses the irregularity in EHR while LSTM performs the downstream task, where NODE and LSTM both are trained in an end-to-end differentiable manner.

**Fig. 7:**
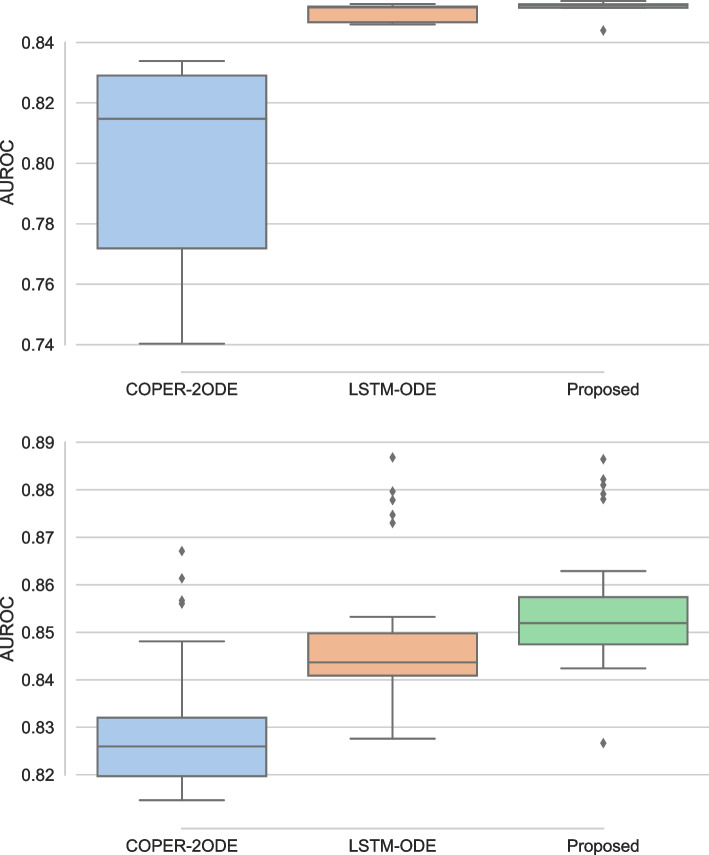
Ablation to study the effect of two Neural ODEs and attention models using MIMIC (left) and Physionet (right) datasets

Figure 7 presents the ablation study for the proposed model using MIMIC and PhysioNet datasets. For simplicity, we present results with AUROC and for 25% missingness as we see similar patterns for AUPRC and other missingness cases. From the figure, we observe similar patterns for both datasets where COPER-2ODE performs worse and shows large variability in performance as compared with the proposed model. This is potentially due to the use of two NODEs where errors in modelling patient state dynamics are exacerbated by the second NODE which learns the dynamics of the hidden state of the model. Since one NODE is sufficient to address irregularity in EHR and learning patient health status provides meaningful interpretation and can be extended to explore disease progression modelling so only one NODE was used in the proposed model. From comparing the proposed model with LSTM-ODE, it is clear that the proposed attention model performs better than LSTM-ODE. LSTM-ODE may be preferable with smaller sequences while the proposed continuous attention models are suitable for longer sequences, which will be explored in the future study.

### Selective predictions and expert referrals

The predictive uncertainty of a machine learning model is useful in guiding the use of a model in high-stake applications, such as healthcare. The models are used for selective predictions and highly uncertain predictions of a model are referred to a clinician for further examination. This will increase the transparency and trust of clinical users in machine learning techniques and will help in the adoption of machine learning in healthcare.

The latents of Perceiver lend a natural support to uncertainty quantification because after processing each of the latent can be passed through separate prediction heads, similar to a multi-task setting, generating multiple predictions. However, we observed that the processed latents at the classifier layer lack diversity and thus fail to express predictive uncertainty. So, ways to learn diversity in the processed latents will be explored in future work. We have used the Monte Carlo (MC) dropout technique [52] for calculating the model’s predictive uncertainty and is an approximation of Bayesian techniques [52] which otherwise are difficult to train. MC dropout is a simple but scalable technique and does not require training multiple models or even retraining models, rather trained models which use dropout for regularisation can be used for uncertainty quantification. MC dropout requires activating the dropout layers during the testing phase, which is otherwise turned off. So, each evaluation of the model with the same data point gives different prediction probabilities. We evaluated our models 25 times on each sample of the test dataset, and the mean and variance of these 25 predictions act as actual predictions and predictive uncertainty of the model.

**Fig. 8:**
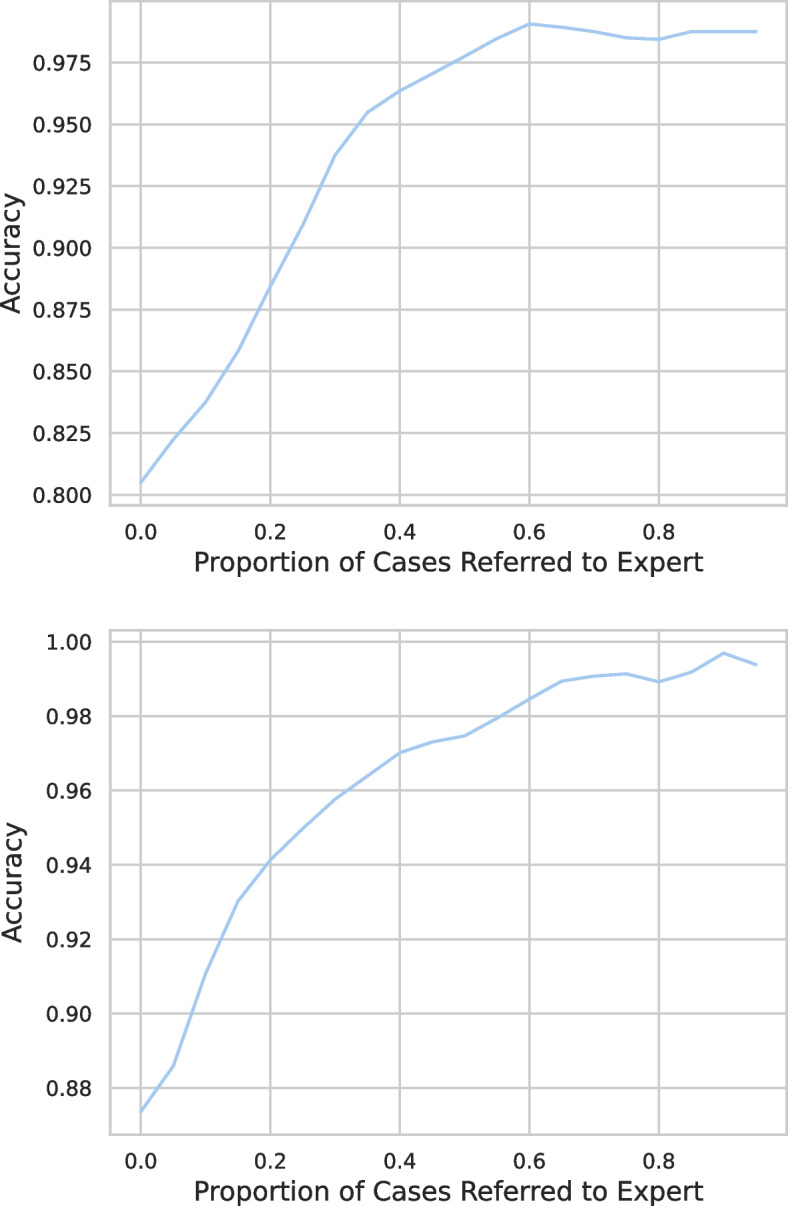
Utilisation of predictive uncertainty for selective predictions and referring the uncertain predictions to the clinicians: The left panel presents the test accuracy of Perceiver on PhysioNet and the right panel presents the test accuracy of the COPER on MIMIC dataset, against the proportion of uncertain cases referred to the clinicians

We refer highly uncertain cases to clinicians and evaluate the model performance selectively on the remaining test dataset. Figure 8 presents test accuracy against the proportion of cases referred to the clinician. The left panel presents the results for Perceiver with PhysioNet and the right panel presents results for COPER with MIMIC dataset at 50% irregularity (selected randomly). Both figures show similar behaviour, and as expected, the performance of both models increases when the uncertain cases are removed. Thus, uncertainty quantification is useful, and cases can be referred to clinicians as per their availability.

## Discussions

Based on the idea of cross-attention-based architectures, we proposed a computationally efficient variant of the transformer, called Perceiver, as a potential alternative for processing time series data in EHR. The cross-attention operation helps to squeeze the long sequences of time series to a smaller number of latents which then can be processed using self-attention operations, requiring fewer computations than directly processing the time series with transformer based models. Perceiver outperforms LSTM and TCN, the widely used techniques for time series, and is at par or better performance than DLinear. Perceiver was able to reduce the computations by around nine times, as compared with transformers with no significant loss of performance. We further extended Perceiver and transformer models to learn the patient health dynamics from the ITS. These continuous models employ neural ordinary differential equations to model patient health trajectory from which patient state can be sampled at any time-step, hence addressing the irregularity issue in EHR.

These continuous attention models can handle long sequences, completely missing time steps, and noisy observations and employ end-to-end learning for handling irregularity. The experiments prove the efficacy of the proposed work on in-hospital-mortality prediction task using PhysioNet and MIMIC-III datasets. Unlike the existing approaches, such as LODE, which model the hidden state dynamics of the neural networks, continuous attention models model patient health state and can be useful for other tasks, like disease progression modelling. We also employ uncertainty quantification for calculating the predictive uncertainty of the proposed models, which was used for selective predictions and referring the uncertain cases to clinicians. This helps in improving the performance of the system, adjusting the working of the models as per the time availability of clinicians, and building transparency and the trustworthiness of the proposed techniques for adoption in healthcare.

LSTM with a carry forward technique for handling irregularity does not perform well as it shows a decrease in performance with the increasing irregularity in EHR. However, Perceiver with carry forward performs significantly better than LSTM with carry forward for handling ITS. Overall, CTransformer outperforms all other techniques. COPER also indicates competitive performance in dealing with irregularity and is computationally less expensive than the CTransformer and LODE models. Amongst all the techniques for handling ITS, LODE is the most expensive and takes up to two days to train on the MIMIC dataset. Thus, the Perceiver and the continuous patient state attention models provide computationally efficient techniques for handling ITS in EHR.

The proposed attention-based models are advanced deep learning models, so they share the same limitations as the other models of the same type, such as requiring more data to train, hyperparameter tuning, and more computational resources than traditional machine learning approaches. Despite this, we were able to reduce computations compared to the transformer, and the proposed models to handle ITS are computationally cheaper than state-of-the-art NODE-based models, such as LODE. To further evaluate the performance of Perceiver and the continuous attention models, in the future, we will study more tasks and datasets across different disciplines, including sepsis prediction in an ICU setting and longer sequences with original cross-attention. Since our work learns the patient’s health dynamics, it could be helpful in disease progression modelling and will be explored in future studies. Moreover, we will explore ways to introduce diversity in the processed latents at the classifier layer for quantifying the predictive uncertainty of the proposed models, because latents provide a natural support for quantifying predictive uncertainty. Additionally, understanding the interpretability of cross-attention layers and comparing them with self-attention layers and other methods of interpretability will be investigated. This analysis will contribute to comprehending how these models capture and utilise information from different parts of the input sequence. Furthermore, future research will explore the scalability of the proposed methods with tasks of varying and longer input sequences, including online handwriting recognition.

## Conclusions

We adapted cross-attention to propose a Perceiver model to process time series in electronic health records. Perceiver, through learnable latents, reduced the computations by nine times as compared with the transformer. To address irregularity in electronic health records, we further propose continuous attention models for Perceiver and transformer which learn a patient’s health-status dynamics. The continuity of the proposed continuous patient state attention models comes from the neural ordinary differential equations which help to sample a patient state at any time-step from observed irregular time-steps. The empirical analysis with in-hospital-mortality task using MIMIC-III and PhysioNet datasets prove the efficacy of the proposed techniques. Moreover, the predictive uncertainty of the model helps in the development of transparent and trustworthy systems, which can be utilised as per the availability of the clinicians.

## Data Availability

The code is freely available and verified at https://codeocean.com/capsule/4587224. Moreover, the datasets are also available from https://physionet.org/content/mimiciii/1.4/ and https://physionet.org/content/challenge-2012/1.0.0/.

## Footnotes

1 https://github.com/YerevaNN/mimic3-benchmarks

2 https://github.com/thuml/Time-Series-Library

3 https://github.com/locuslab/TCN

4 https://github.com/reml-lab/mTAN

5 https://github.com/YuliaRubanova/latent_ode

6 https://github.com/thuml/Time-Series-Library
